# Low pre-ART CD4 count is associated with increased risk of clinical progression or death even after reaching 500 CD4 cells/μL on ART

**DOI:** 10.1371/journal.pone.0283648

**Published:** 2023-03-30

**Authors:** Nikos Pantazis, Vasilios Paparizos, Vasilios Papastamopoulos, Simeon Metallidis, Anastasia Antoniadou, Georgios Adamis, Mina Psichgiou, Maria Chini, Helen Sambatakou, Georgios Chrysos, Nikolaos V. Sipsas, Charalambos Gogos, Emmanouil Barbunakis, Periklis Panagopoulos, Olga Katsarou, Giota Touloumi, on behalf of the Athens Multicenter AIDS Cohort Study (AMACS)

**Affiliations:** 1 Dept. of Hygiene, Epidemiology and Medical Statistics, Medical School, National and Kapodistrian University of Athens, Athens, Greece; 2 AIDS Unit, Clinic of Venereologic & Dermatologic Diseases, Medical School, Syngros Hospital, National and Kapodistrian University of Athens, Athens, Greece; 3 Division of Infectious Diseases, 5^th^ Department of Internal Medicine, Evangelismos General Hospital of Athens, Athens, Greece; 4 1^st^ Internal Medicine Department, Infectious Diseases Unit, AHEPA University Hospital, Aristotle University of Thessaloniki, Thessaloniki, Greece; 5 4^th^ Dept. of Internal Medicine, Medical School, National and Kapodistrian University of Athens, Attikon University General Hospital, Athens, Greece; 6 1^st^ Dept. of Internal Medicine and Infectious Diseases Unit, General Hospital of Athens G. Gennimatas, Athens, Greece; 7 1^st^ Dept. of Internal Medicine, Medical School, National and Kapodistrian University of Athens, Athens, Greece; 8 3^rd^ Dept. of Internal Medicine Infectious Diseases Unit, Red Cross General Hospital, Athens, Greece; 9 2^nd^ Dept. of Internal Medicine, HIV Unit, National and Kapodistrian University of Athens, Medical School, Hippokration University General Hospital, Athens, Greece; 10 Infectious Diseases Unit, Tzaneion General Hospital of Piraeus, Athens, Greece; 11 Department of Pathophysiology, Infectious Diseases Unit, Medical School, Laikon Athens General Hospital and National and Kapodistrian University of Athens, Athens, Greece; 12 Dept. of Internal Medicine & Infectious Diseases, Patras University General Hospital, Patras, Greece; 13 Dept. of Internal Medicine, University Hospital of Heraklion, Heraklion, Crete, Greece; 14 2nd University Department of Internal Medicine, Infectious Diseases Unit, University Hospital of Alexandroupolis, Democritus University of Thrace, Alexandroupolis, Greece; 15 Blood Centre, National Reference Centre for Congenital Bleeding Disorders, Laikon Athens General Hospital and Medical School, National and Kapodistrian University of Athens, Athens, Greece; University Hospital Zurich, SWITZERLAND

## Abstract

**Introduction:**

Clinical disadvantages of initiating ART at low CD4 counts have been clearly demonstrated but whether any excess risk remains even after reaching relatively high/safe CD4 levels remains unclear. We explore whether individuals starting ART with <500 CD4 cells/μL who increased their CD4 count above this level, have, from this point onwards, similar risk of clinical progression to serious AIDS/non-AIDS events or death with individuals starting ART with ≥500 CD4 cells/μL.

**Methods:**

Data were derived from a multicenter cohort (AMACS). Adults, starting PI, NNRTI or INSTI based ART, in or after 2000 were eligible, provided they started ART with ≥500 (“High CD4”) or started with CD4 <500 cells/μL but surpassed this threshold while on ART (“Low CD4”). Baseline was the date of ART initiation (“High CD4”) or of first reaching 500 CD4 cells/μL (“Low CD4”). Survival analysis, allowing for competing risks, was used to explore the risk of progression to study’s endpoints.

**Results:**

The study included 694 persons in the “High CD4” and 3,306 in the “Low CD4” group. Median (IQR) follow-up was 66 (36, 106) months. In total, 257 events (40 AIDS related, 217 SNAEs) were observed. Rates of progression did not differ significantly between the two groups but the subgroup of those initiating ART with <200 CD4 cells/μL had significantly higher risk of progression after baseline, compared to those in the “High CD4” group.

**Conclusions:**

Individuals starting ART with <200 cells/μL remain on increased risk even after reaching 500 CD4 cells/μL. These patients should be closely followed.

## Introduction

The introduction of combined antiretroviral therapy (ART) [1, 2], along with its continuous efficacy and tolerability improvements, has transformed HIV into a controllable, chronic disease [3–5].

The primary goal of ART is to achieve sustained viral suppression and preserve or restore the immunologic function [6, 7]. Initiating ART early during the course of the infection has been associated with favourable immunologic outcomes [8, 9]. However, initial concerns regarding development of resistance and toxicity, led to a conservative approach with ART’s initiation being delayed until CD4 cell count decline below certain thresholds. Nowadays though, ART is recommended irrespective of CD4 cell count [6, 7], mainly due to the results of two critical trials [10, 11]; both the TEMPRANO [10] and the START [11] trial demonstrated that immediate initiation of ART, with CD4 cell count above 500 cells/μl, was associated with decreased risk of death, severe HIV-related illnesses and serious non-AIDS related events.

Although the clinical disadvantages of initiating ART at low CD4 counts have been clearly demonstrated [12, 13], a substantial proportion of HIV infected individuals start treatment with CD4 cell counts well below 500 cells/μl, mainly due to late presentation to care [14, 15]. Fortunately, current ART regimens usually lead to sustained viral suppression and substantial CD4 cell count increases even among persons starting treatment with very low CD4 counts (e.g. <200 cells/μl) [16, 17].

Guidelines, regarding clinical monitoring and care of persons on ART, depend on the degree and duration of viral suppression as well as the degree of immunologic response. USA guidelines suggest that, for persons with sustained viral suppression and CD4 count above 500 cells/μl, viral load monitoring can be extended to every 6 months and CD4 monitoring is optional while they refer to the >500 CD4 cells/μl range as “normal” [6]. European guidelines, suggest annual CD4 monitoring for those on stable and suppressive ART with CD4 counts >350 cells/μl [7].

The association of poor immunologic response to treatment with increased risk of both AIDS and non-AIDS related events is well established [18–20]. However, it is not clear whether the higher risk of clinical progression associated with low CD4 counts at treatment initiation, remains even after a satisfactory immunologic response to CD4 levels above a level which is considered relatively safe (i.e. 500 cells/μl).

Our objective was to compare the rate of progression to serious AIDS or non-AIDS events observed after CD4 restoration to at least 500 cells/μl, among persons who had initiated ART with <500 CD4 cells/μl, with the corresponding rate observed after ART initiation among persons who initiated ART with ≥500 CD4 cells/μl. Additionally we will investigate if there is any dose-response relationship in the above comparison by further stratifying the group of those initiating ART with <500 CD4 cells/μl in subgroups according to their CD4 cell count at ART initiation. We hypothesized that the association of lower pre-ART CD4 levels with higher risk for clinical progression persists even after adequate immunologic response to ART. More specifically, our scientific hypothesis is that individuals who started ART with less than 500 CD4 cells/μl and subsequently restored them to levels above 500 CD4 cells/μl continue to be at higher risk for clinical progression relative to persons who started ART with a well preserved immune system (i.e. with more than 500 CD4 cells/μl). Evidence in favour of this hypothesis would imply that individuals starting ART with low CD4 levels should be cautiously monitored even after adequate CD4 reconstitution.

## Materials and methods

### Study design and participants

Data were derived from the Athens Multicenter AIDS Cohort Study (AMACS) updated at the end of 2019. AMACS is a collaborative, open, ongoing, population-based cohort study initiated in 1996. Currently, 14 of the 16 HIV-1 clinics in Greece participate in the study. More details about the AMACS study are provided elsewhere [16]. The study protocol was reviewed and approved by the Hellenic Centre for Diseases Control and Prevention, the National Organization for Medicines, the Athens University Medical School Ethics committee, and by the corresponding hospital’s scientific committee of each participating clinic.

For the current study, all adults (≥18 years old) who initiated a ART regimen based on boosted protease inhibitors (bPIs), non-nucleoside reverse transcriptase inhibitors (NNRTIs) or integrase strand transfer Inhibitors (INSTIs) in 2000 or later, while previously antiretroviral therapy naïve, were eligible. We excluded individuals without CD4 cell count or HIV-RNA viral load (VL) measurements within 6 months prior to ART initiation. We also excluded individuals who initiated ART with <500 CD4 cells/μl and never reached CD4 levels of ≥500 cells/μl later on. Finally we excluded those with less than 3 months of follow-up after the baseline date (see Definitions sub-section below).

### Definitions

Eligible individuals were classified into two groups according to their CD4 cell count at ART initiation: a) Those who started ART with ≥500 CD4 cells/μl (“High CD4” group) and b) Those who started ART with <500 CD4 cells/μl but reached CD4 levels of ≥500 cells/μl later on (“Low CD4” group). The “Low CD4” group was further divided by CD4 cell count at ART initiation (<200, 200–349, 350–499 cells/μL).

**Fig 1:**
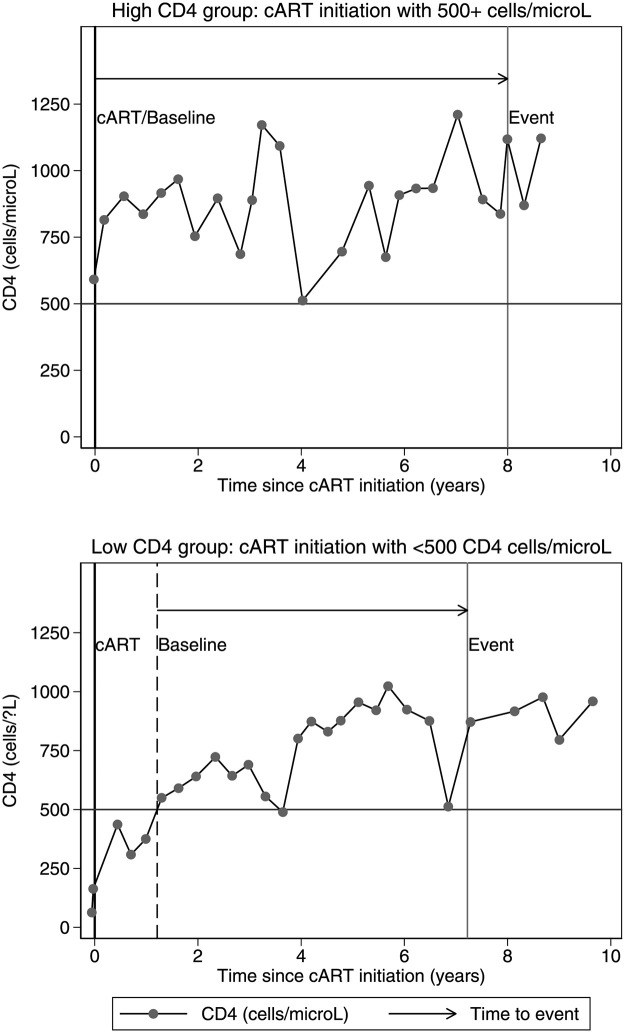
Illustration of the definition of baseline date (as a time origin for the time-to-event analyses) using data from one patient in the “High” and one in the “Low” CD4 group.

For the “High CD4” group the date of ART initiation was considered as the baseline date. For individuals in the “Low CD4” group, the baseline date was estimated as the date they reached 500 CD4 cells/μl for the first time after ART initiation. Estimation of this date was based on a linear interpolation of CD4 cell count between the last time it was below 500 and the first time it was ≥500 cells/μl (Fig 1).

### Endpoints

The primary endpoint of the study is the development of a serious AIDS event, a serious non-AIDS event (SNAE), or death from any cause (AIDS or non-AIDS or of unknown cause). Serious AIDS events include all traditional (i.e. those in the 1993 CDC expanded surveillance definition) opportunistic infections or malignancies excluding oesophageal candidiasis and chronic herpes simplex infection. Serious AIDS events within the first 3 months after ART initiation have been ignored. SNAEs include cardiovascular disease (CVD; i.e. myocardial infarction, stroke, coronary revascularization), end stage renal disease (ESRD; i.e. initiation of dialysis, renal transplantation), decompensated liver disease and non-AIDS defining malignancies (NADM) excluding basal and squamous cell skin cancers.

Secondary endpoints resulted from the classification of the primary endpoints into AIDS related ones (serious AIDS events and AIDS related deaths) and SNAE related ones (SNAE and non-AIDS deaths). Additional secondary endpoints resulted from further stratification of the SNAE related ones into those related to CVD, NADM or other conditions and regrouping of events into fatal and non-fatal ones. Finally, death from any cause, without censoring at the occurrence of a previous non-fatal event, was also considered as a secondary endpoint.

### Statistical analysis

Demographic and clinical characteristics were summarized (overall and by CD4 group) using standard procedures. More specifically, for categorical variables absolute (N) and relative (%) frequencies are provided and between groups comparisons were based on exact tests for categorical data. Normally distributed continuous variables are summarized through means and standard deviations and compared using t-tests. Finally for non-normally distributed continuous variables medians, interquartile ranges and Mann-Whitney U-tests are used.

Main analyses were based on time-to-event methods. For the analysis of the primary endpoint and the all cause mortality, Kaplan-Meier failure curves, log-rank tests and Cox proportional hazards models were used. Incidence rates were calculated by dividing the number of failures by the person-time. Respective confidence intervals were calculated using the quadratic approximation to the Poisson log likelihood for the log-rate parameter. For the analysis of each of the remaining endpoints, methods for the analysis of competing risks were used. For example, for the analysis of the AIDS related events, SNAE related ones were considered as a competing risk and vice-versa. In the competing risks analyses, curves of the cumulative incidence function (CIF) [21], Gray’s tests [22] and Fine and Gray models [23] were used.

Besides CD4 at ART initiation, other covariates considered in multivariable models included sex, age at ART initiation, risk group, HIV-RNA viral load at ART initiation, class of ART regimen (i.e. based on boosted PI, NNRTI or INSTI), calendar year of ART initiation, pre-ART AIDS diagnosis and degree of virologic suppression. Degree of virologic suppression was expressed as the percentage of follow-up time (excluding the first three months of ART) with undetectable (i.e. <50 copies/mL) HIV-RNA viral load. This quantity was used as a proxy for the virologic success or failure of treatment of each patient averaged over his/her whole follow-up time.

Power calculations through simulations [24] showed that, given the current study’s characteristics (sample size, number of events, CD4 distribution at ART initiation, rate of progression to the primary outcome) the power to detect differences between the four CD4 subgroups (through a global Wald test with 3 degrees of freedom) similar to those estimated in the study (i.e. same to the adjusted hazard ratios for the <200, 200–349 and 350–499 CD4 cells/μl subgroups compared to the 500+ CD4 cells/μl group; see Table 2) or lower (log hazard ratios reduced by 20%), at an alpha level of 0.05, was 93.8% or 79.3%, respectively.

P-values less than 0.05 were considered statistically significant. All analyses have been performed using Stata 14.2 [25] and R 4.0.2. [26]

## Results

Of the 11,658 individuals in AMACS data base, 5,318 started a boosted PI, NNRTI or INSTI based ART regimen, in or after 2000, while at least 18 years old and had CD4 cell count and HIV-RNA measurements available close to ART initiation. Of them, 720 started ART with ≥500 CD4 cells/μl and 4,598 with <500 CD4 cells/μl. Of the latter, 1,244 individuals were excluded as they did not reach ≥500 CD4 cells/μl after ART initiation. Of the remaining 4,074 individuals, 4,000 (694 “High CD4” and 3,306 in “Low CD4” group) had ≥3 months of follow-up after the baseline date (S1 Fig).

Demographic and clinical characteristics of the study sample are shown in Table 1. Most of the study participants were men (87.5%) and infected through sex between men (65.6%) but these percentages were lower in the “Low CD4” group. Individuals in the “High CD4 group” tended to be younger (36.4 *vs*. 37.5 years) and with lower VL at ART initiation (4.2 *vs*. 4.6 log_10_ copies/mL) compared to those in the “Low CD4” group. A small proportion (6.6%) of the study participants had already progressed to clinical AIDS before initiating ART and an even smaller one (1.4%) developed AIDS soon after treatment’s initiation. In both cases, percentages were significantly higher in the “Low CD4” group. In general, the “Low CD4” group is comprised of individuals who were diagnosed and treated in earlier years (median diagnosis year: 2009 *vs*. 2012; median ART initiation year: 2011 *vs*. 2014) compared to the “High CD4” group, with this difference being also reflected in the type of their treatment (i.e. based mainly on boosted PIs rather than INSTIs or NNRTIs). Their median CD4 count at ART initiation was 283 cells/μl and it took approximately 7 months to increase it to levels above 500 cells/μl. Due to their treatment initiation in earlier years, their follow-up in this study was longer (70 vs. 50 months). Finally individuals in the “Low CD4” group seemed to have higher proportions of follow-up time with undetectable viral load compared to those in the “High CD4” group.

**Table 1:** Demographic and clinical characteristics by CD4 at ART initiation and overall. Figures are n (%), Mean (SD) or Median (IQR).

	Group			
	High CD4^1^	Low CD4^2^	Overall	p-value
	*n = 694 (17*.*3%)*	*n = 3306 (82*.*7%)*	*N = 4000 (100%)*	
Sex				0.005*
*Male*	629 (90.6%)	2870 (86.8%)	3499 (87.5%)	
*Female*	65 (9.4%)	436 (13.2%)	501 (12.5%)	
Age at ART initiation (yrs)	36.4 (9.8)	37.5 (10.2)	37.3 (10.1)	0.007^§^
Risk group				<0.001*
*MSM*^3^	498 (71.8%)	2126 (64.3%)	2624 (65.6%)	
*IDU*^4^	57 (8.2%)	243 (7.4%)	300 (7.5%)	
*Heterosexual*	98 (14.1%)	701 (21.2%)	799 (20.0%)	
*NA*	41 (5.9%)	236 (7.1%)	277 (6.9%)	
CD4 (cells/μL) at ART initiation	631 (550, 765)	283 (190, 355)	312 (215, 426)	<0.001^Ŧ^
HIV-RNA (log_10_ copies/mL) at ART initiation	4.2 (1.1)	4.6 (0.9)	4.5 (1.0)	<0.001^§^
Pre-ART AIDS history				<0.001*
*None*	679 (97.8%)	3000 (90.7%)	3679 (92.0%)	
*Before ART*	11 (1.6%)	253 (7.7%)	264 (6.6%)	
*Within 3 months of ART*	4 (0.6%)	53 (1.6%)	57 (1.4%)	
AIDS before baseline^5^				<0.001*
*No*	682 (98.3%)	2932 (88.7%)	3614 (90.3%)	
*Yes*	12 (1.7%)	374 (11.3%)	386 (9.7%)	
Type of ART				<0.001*
*NNRTI*^6^	290 (41.8%)	1401 (42.4%)	1691 (42.3%)	
*INSTI*^7^	222 (32.0%)	433 (13.1%)	655 (16.4%)	
*Boosted PI*^8^	182 (26.2%)	1472 (44.5%)	1654 (41.3%)	
Calendar year of ART initiation				<0.001*
*2000–04*	55 (7.9%)	358 (10.8%)	413 (10.3%)	
*2005–09*	85 (12.2%)	1008 (30.5%)	1093 (27.3%)	
*2010–14*	268 (38.6%)	1414 (42.8%)	1682 (42.0%)	
*≥2015*	286 (41.2%)	526 (15.9%)	812 (20.3%)	
ART initiation to baseline^5^ (months)		6.8 (2.0, 19.5)		
F-Up since baseline^5^ (months)	50 (28, 86)	70 (39, 108)	66 (36, 106)	<0.001^Ŧ^
% of F-Up time with undetectable Viral Load				<0.001*
*0–29*	105 (15.1%)	51 (1.5%)	156 (3.9%)	
*30–69*	111 (16.0%)	192 (5.8%)	303 (7.6%)	
*70–89*	81 (11.7%)	415 (12.6%)	496 (12.4%)	
*≥90*	397 (57.2%)	2366 (71.6%)	2763 (69.1%)	
*NA*	0 (0.0%)	282 (8.5%)	282 (7.0%)	

^1^: Started ART with ≥500 CD4 cells/μl;

^2^: Started ART with <500 CD4 cells/μl but reached at least 500 CD4 cells/μl later on;

^3^: Men having sex with men;

^4^: Intravenous drug use;

^5^: Date of ART initiation (High CD4 group) or date of first reaching 500 CD4 cells/μl (Low CD4 group);

^6^: Non-nucleoside reverse transcriptase inhibitors;

^7^: Integrase strand transfer inhibitors;

^8^: Protease Inhibitors.

* exact test for categorical data;

^§^ t-test;

^Ŧ^ Mann-Whitney U-test

Number of events (only the first event considered in cases of individuals with multiple events) occurring between the baseline date and end of follow-up, by CD4 group, are given in Table 2. In this table, and in subsequent analyses, individuals in the “Low CD4” group (n = 3,306) are also further divided into 3 subgroups according to their CD4 count at ART initiation: a) 350–499 cells/μl (n = 887; 26.8%), b) 200–349 cells/μl (n = 1,529; 46.3%) and c) <200 cells/μl (n = 890; 26.9%).

**Table 2:** Events [n (%)] observed between baseline date and end of follow-up by CD4 group and overall. “Low CD4” group stratified to subgroups. All numbers refer to the first event observed during follow-up (in case of multiple events/individual).

	High CD4^1^	Low CD4^2^	Low CD4^2^ (subgroups)		
	(n = 694)	(n = 3,306)	350–499 CD4/μl (n = 887)	200–349 CD4/μl (n = 1,529)	<200 CD4/μl (n = 890)
**Type of event—detailed**					
*Serious AIDS*	6	29	5	12	12
	(0.86%)	(0.88%)	(0.56%)	(0.78%)	(1.35%)
*AIDS death*	1	4		2	2
	(0.14%)	(0.12%)		(0.13%)	(0.22%)
*CVD*^3^	10	54	13	17	24
	(1.44%)	(1.63%)	(1.47%)	(1.11%)	(2.70%)
*CVD*^3^ *death*	1	22	3	12	7
	(0.14%)	(0.67%)	(0.34%)	(0.78%)	(0.79%)
*ESRD*^4^ *death*		2			2
		(0.06%)			(0.22%)
*Liver decompensation*		1		1	
		(0.03%)		(0.07%)	
*NADM*^5^	7	45	14	17	14
	(1.01%)	(1.36%)	(1.58%)	(1.11%)	(1.57%)
*NADM*^5^ *death*		10	3	2	5
		(0.30%)	(0.34%)	(0.13%)	(0.56%)
*Death-other causes*	11	54	7	22	25
	(1.59%)	(1.63%)	(0.79%)	(1.44%)	(2.81%)
**All events (primary endpoint)**	36	221	45	85	91
	(5.19%)	(6.68%)	(5.07%)	(5.56%)	(10.22%)

^1^: Started ART with ≥500 CD4 cells/μl;

^2^: Started ART with <500 CD4 cells/μl but reached at least 500 CD4 cells/μl later on;

^3^: Cardiovascular disease;

^4^: End stage renal disease;

^5^: Non-AIDS malignancies; 6: Serious non-AIDS event

In total, 130 (5.2/1000 person-years) individuals died during follow-up with the corresponding numbers (rates) being 16 (4.3), 19 (3.7), 43 (4.2) and 52 (8.9) for those who started ART with 500+, 350–499, 200–349 and <200 CD4 cells/μl, respectively.

### Analysis of primary endpoint

**Fig 2:**
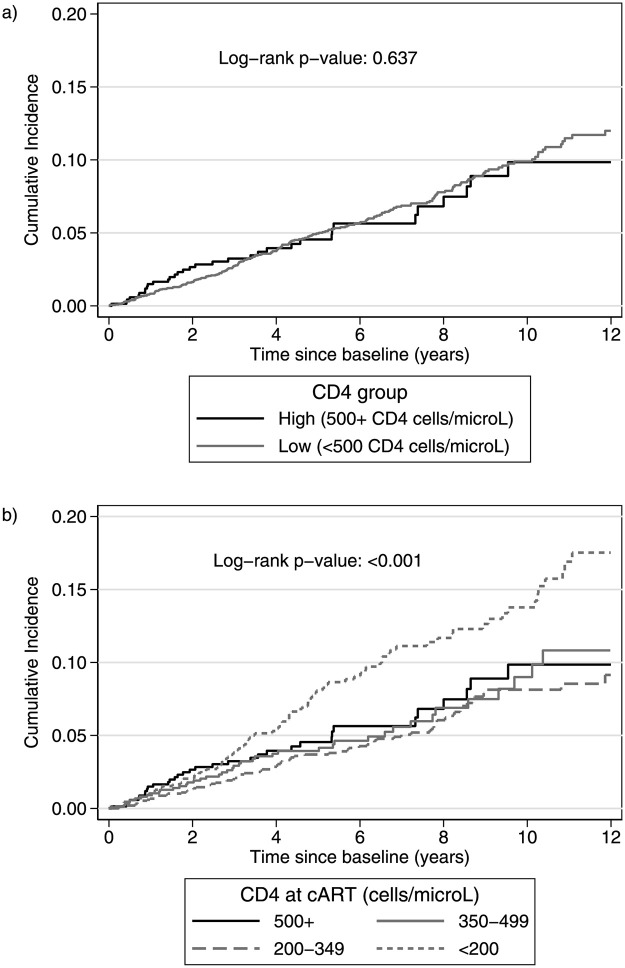
Cumulative probability of primary endpoint (Serious AIDS, Serious non-AIDS or death from any cause) by a) CD4 group (“High” i.e. those who started ART with 500+ and “Low” i.e. those who started ART with <500 CD4 cells/μl) and b) CD4 group with further stratification of the “Low” CD4 group according to CD4 at ART initiation. Baseline is the date of ART initiation (“High” CD4 group) or the date of first reaching 500 CD4 cells/μl (“Low” CD4 group).

Cumulative probabilities of progression to the primary endpoint (serious AIDS, SNAE or death from any cause) for the two main CD4 groups and with further stratification of the “Low CD4” group are presented in Fig 2a and 2b, respectively. Unadjusted comparisons, showed that the difference in the primary endpoint rates, between the “High” and “Low” CD4 groups, was not statistically significant (log-rank p = 0.637). However, individuals starting ART with CD4 count below 200 cells/μl, have higher rates of reaching the primary endpoint after the baseline compared to all other groups (log-rank p<0.001).

Results of univariable and multivariable Cox models for the hazard of the primary endpoint are shown in Table 3. As shown in this table, the difference between the “Low CD4” group and the “High CD4” group was only suggestive (adjusted Hazard Ratio 1.37; p = 0.097) of slightly higher risk of progression for the latter. Further stratification of the “Low CD4” group revealed a trend for higher risk at lower CD4 levels at ART initiation. The difference between the “350–499 CD4/μl” or “200–349 CD4/μl” subgroups with the “High CD4” (500+ CD4/μl) group was clearly non-significant (p = 0.539 and 0.400, respectively). However, those who started ART with very low CD4 cell count (i.e. <200 cells/μl) had double the risk of progression to the primary endpoint compared to the “High CD4” group (adjusted Hazard Ratio 1.99; p = 0.001). Regarding the remaining factors in this multivariable model, higher age, IDU risk group and smaller percentage of follow-up with viral suppression were significantly associated with higher risk of progression. Similar, but marginally non significant, associations were found for male sex and initiation of ART in earlier years whereas class of ART regimen, HIV-RNA viral load at ART initiation and pre-ART AIDS diagnosis were not significantly associated with the risk of progression given the remaining factors in the multivariable model (data not shown).

**Table 3:** Results of a univariable and multivariable Cox models for the hazard of the primary endpoint (serious AIDS, serious non-AIDS or death from any cause).

Group (CD4 at ART initiation cells/μl)	Number of HIV+ adults on ART (%of total), n = 4000	Number (%) with primary endpoint	Time at risk (person-years)	Incidence rate (95% CI)	Crude HR (95% CI; p-value)	Adjusted* HR (95% CI; p-value)
High (500+) [reference]	694 (17.35)	36 (5.19)	3649.5	9.86 (7.12–13.68)	Ref	Ref
Low (<500)	3306 (82.65)	221 (6.68)	20639.6	10.71 (9.39–12.22)	1.09 (0.76–1.55; 0.637)	1.37 (0.94–1.98; 0.097)
High (500+) [reference]	694 (17.35)	36 (5.19)	3649.5	9.86 (7.12–13.68)	Ref	Ref
350–499	887 (22.18)	45 (5.07)	4975.3	9.04 (6.75–12.11)	0.92 (0.60–1.43; 0.725	1.15 (0.74–1.80; 0.539)
200–349	1,529 (38.23)	85 (5.56)	10003.3	8.50 (6.87–10.51)	0.87 (0.59–1.28; 0.468	1.19 (0.79–1.79; 0.400)
<200	890 (22.25)	91 (10.22)	5661.0	16.08 (13.09–19.74)	1.63 (1.10–2.39; 0.014	1.99 (1.31–3.01; 0.001)

Hazard ratios (HR) were estimated using Cox proportional hazards model;

*estimates adjusted for age at ART initiation, sex, risk group, % of F-Up time with undetectable Viral Load and year of ART initiation

### Analysis of secondary endpoints

S2–S4 Figs show cumulative incidence function curves from competing risks analyses of a) serious AIDS or AIDS death *vs*. SNAE or non-AIDS death b) fatal vs. non-fatal events and c) serious AIDS or AIDS death vs. CVD vs. NADM related events vs. all other types of events, respectively. S5 Fig shows cumulative probabilities of death from any cause (without any competing risk and without censoring at the occurrence of any other event).

All secondary endpoints analyses show similar patterns to those observed in the primary endpoint analysis. More specifically, differences in event rates between the “High CD4” group and subgroups of the “Low CD4” group comprising those who started ART with 200–499 CD4 cell counts/μl seem negligible. However, those who started ART with <200 CD4 cells/μl seem to have increased rates, compared to the “High CD4” group in several cases: unadjusted comparisons, allowing for competing risks, showed significantly increased rates of SNAEs or non-AIDS deaths (p = 0.001), fatal events (p = 0.001) and non-AIDS/CVD/NADM related events (mainly deaths from other causes; p = 0.009). Similar results were found for the overall mortality (p<0.001).

Adjusted Hazard ratios (from multivariable Cox model for the overall mortality) or sub-distribution Hazard Ratios (from Fine and Gray models for all other secondary endpoints) are provided in Table 4. As shown in this table, differences between those in the “Low CD4” group who started ART with 350–499 or 200–349 CD4 cells/μl and those in the “High CD4” group were small and statistically non-significant. However, those who started ART with <200 CD4 cells/μl had significantly higher rates compared to those in the “High CD4” group in most cases. The most pronounced differences were observed in the competing risks analysis of serious AIDS and AIDS related deaths (adjusted sub-distribution Hazard Ratio [asHR] = 3.60; p = 0.018) and in the analysis of overall mortality (adjusted Hazard Ratio = 2.44; p = 0.004). For sub-categories of SNAEs and SNAE related deaths (CVD, NADM, Other), asHRs ranged from 1.62 to 1.78 and were not statistically significant, but for all SNAEs and non-AIDS deaths combined, the corresponding asHR was 1.77 and statistically significant (p = 0.011). Finally, the analysis of all fatal events with non-fatal ones as competing risk resulted in a significant (p = 0.016) asHR of 2.20 and the same holds for the analysis of non-fatal events with fatal ones as competing risk (asHR = 1.78; p = 0.038).

**Table 4:** Results from multivariable Fine and Gray models (when competing risk is present) and Cox proportional hazards model (for overall mortality).

CD4 at ART initiation groups	aHR*	95% C.I.	p-value
Serious AIDS or AIDS death (competing risk: SNAE or non-AIDS death)			
*350-499/500+*	1.10	(0.34, 3.52)	0.871
*200-349/500+*	1.92	(0.71, 5.24)	0.201
*<200/500+*	3.60	(1.25, 10.36)	0.018
SNAE or non-AIDS death (competing risk: serious AIDS or AIDS death)			
*350-499/500+*	1.16	(0.71, 1.89)	0.558
*200-349/500+*	1.06	(0.68, 1.64)	0.809
*<200/500+*	1.77	(1.14, 2.74)	0.011
CVD or CVD death (competing risk: all other incl. AIDS/AIDS death)			
*350-499/500+*	1.20	(0.54, 2.66)	0.656
*200-349/500+*	1.11	(0.54, 2.29)	0.771
*<200/500+*	1.75	(0.88, 3.50)	0.112
NADM or NADM death (competing risk: all other incl. AIDS/AIDS death)			
*350-499/500+*	1.78	(0.74, 4.31)	0.198
*200-349/500+*	1.07	(0.44, 2.59)	0.881
*<200/500+*	1.78	(0.72, 4.39)	0.209
non-AIDS/CVD/NADM related events (competing risk: all other incl. AIDS/AIDS death)			
*350-499/500+*	0.63	(0.25, 1.61)	0.334
*200-349/500+*	0.98	(0.48, 2.01)	0.963
*<200/500+*	1.62	(0.80, 3.31)	0.183
Fatal events (competing risk: non-fatal events)			
*350-499/500+*	0.96	(0.43, 2.14)	0.913
*200-349/500+*	1.44	(0.76, 2.73)	0.269
*<200/500+*	2.20	(1.16, 4.18)	0.016
Non-Fatal events (competing risk: fatal events)			
*350-499/500+*	1.27	(0.73, 2.19)	0.396
*200-349/500+*	1.04	(0.61, 1.75)	0.894
*<200/500+*	1.78	(1.03, 3.06)	0.038
Overall mortality (no other censoring/competing risks)			
*350-499/500+*	1.12	(0.57, 2.20)	0.749
*200-349/500+*	1.33	(0.73, 2.43)	0.344
*<200/500+*	2.44	(1.34, 4.45)	0.004

*: Hazard Ratios (overall mortality) and sub-distribution Hazard Ratios (all other outcomes) for subgroups of the “Low CD4” group vs. “High CD4” (i.e. >500 cells/μl) group, adjusted (for age at ART initiation, sex, risk group, % of F-Up time with undetectable Viral Load, year of ART initiation).

## Discussion

In this study we investigated whether those who initiated ART having CD4 counts below 500 cells/μl remained at higher risk of progression to serious clinical events or death even after having increased their CD4 counts above 500 cells/μl compared to those who initiated ART having CD4 counts 500 cells/μl or more. The analysis period for the former group included their follow-up time after reaching 500 CD4 cells/μl for the first time after ART initiation while for the second group since ART initiation.

Our results showed that, although rates of progression to all study endpoints did not differ between these two groups, further stratification (based on the CD4 levels at ART initiation) revealed significantly higher rates of progression for those who started ART with <200 CD4 cells/μl and reached ≥500 CD4 cells/μl later on, compared those who started ART having CD4 cells ≥500 CD4 cells/μl. Despite their adequate immunologic response to ART, these individuals had, after reaching a CD4 cell count above 500 cells/μl, double the risk of progression to the study’s primary endpoint (serious AIDS events, serious non-AIDS events or death) compared to those who started treatment prior to their CD4 declining below 500 cells/μl. Similarly, their overall mortality was almost 2.5 times higher, serious AIDS events or AIDS death 3.6 times and serious non-AIDS events or non-AIDS death 1.8 times higher. Differences in the rates of progression to specific categories of SNAEs did not reach nominal statistical significance levels but their direction and magnitude were comparable to those observed when combining all SNAEs categories. Additionally, in most endpoints’ analyses, differences between subgroups of the <500 CD4 cells/μl group with the ≥500 CD4 cells/μl at ART initiation group showed a trend towards higher risk with lower CD4 counts at ART initiation.

Our findings highlight important aspects of the provided care and monitoring of individuals who initiate treatment late and especially for those with less than 200 CD4 cells/μl: although many patients of this specific group may achieve both virologic suppression and adequate immunologic response to CD4 counts (above 500 cells/μl), their increased risk of clinical progression does not return to the lower levels seen among those who initiate treatment with a well preserved immune system. Therefore, a more frequent and more intense monitoring may be needed in this specific group.

The negative association of both AIDS and non-AIDS related morbidity and mortality with CD4 cell count levels at ART initiation is well established by many studies [10, 11, 13, 27]. Low pre-ART CD4 counts are also associated with lower likelihood of CD4 normalization [16, 28–31]. The significance of low CD4 count as a predictor of clinical progression seems to persist even while on viral suppressive ART with several studies showing increased rates among those with poor CD4 response [32–36].

However, it is not clear whether the risk of major morbidity and mortality due to delayed ART initiation persists after restoring CD4 counts to levels above 500 cells/μl. May et al. [37] found that the mortality of persons who started ART with low CD4 count converged with the mortality of those with higher baseline CD4 counts after surviving 5 years of treatment but those who started ART with <200 CD4 cells/μl had higher mortality rates during years 5–10 on ART compared to those with more than 500 CD4 cells/μl at baseline. Mocroft et al. [38] analyzed data from 33 European cohorts within the COHERE collaboration and found increased incidence of AIDS-defining illnesses among those on ART with a current CD4 count of 500–749 cells/μl compared to those with higher CD4 levels suggesting that immune reconstitution is not complete until the CD4 increases to >750 cells/μL.

Our results, although not directly comparable with those of the aforementioned studies, are not inconsistent with them. The most plausible explanation for the increased risk of clinical progression, persisting even after restoration of CD4 count to >500 cells/μl, found in our analyses, is that persons who initiate ART at very low CD4 counts (i.e. <200 cells/μl) have a long prior period of uncontrolled viral replication resulting in increased exposure to activated inflammatory and coagulation pathways [39, 40]. Increased levels of activation and coagulation biomarkers have been associated with increased all-cause mortality [41] and opportunistic infections [42]. Additionally, the immunologic damage in the low CD4 group, due to the prolonged period between infection and treatment, is likely to persist despite their increase in CD4 cell counts. For example it has been shown that phenotypic alterations in peripheral blood natural killer cells induced by ART do not result in improved targeting of HIV [43] and pre-existing immunological memory does not recover despite substantial post-ART CD4 cell count increases [44].

There are several limitations to our study. Although all models were adjusted for potential confounders such as gender, age, route of transmission, calendar year and degree of virologic suppression, residual confounding (e.g. due to lifestyle characteristics) cannot be ruled out as in most observational studies. Additionally, the exact time of crossing the 500 CD4 cells/μl threshold for the low CD4 group was not known and was estimated through interpolation. Due to the time period considered in our study and the persisting rates of late presentation, the group of those who initiated treatment with CD4 counts above 500 cells/μl included fewer individuals with shorter follow-up times compared to those who started treatment with lower CD4 levels. Finally, the high CD4 group had a minimum of 500 CD4 cells/μl at baseline (which coincided with date of ART initiation for this group) whereas all individuals in the low CD4 group had an estimated level of 500 CD4 cells/μl at baseline (by definition of this group) which resulted in a difference of approximately 130 CD4 cells/μl at baseline in favour of the former.

## Conclusions

Despite the potential shortcomings of our study, we believe that our findings carry an important message regarding both, the care and monitoring of HIV infected individuals who commence therapy late and the public health policy in the HIV field. Individuals who initiate treatment having low CD4 counts, especially those with less than 200 CD4 cells/μl, require additional attention not only until they increase their CD4 count to levels considered as safe (i.e. 500 cells/μl) but also after that point as they, most likely, are still at increased risk of both AIDS and non-AIDS serious events including death. On the other hand, our results underline the importance of timely diagnosis and treatment as they suggest that the increased risk of clinical progression or death associated with low CD4 counts at ART initiation persists for much longer than the time required to achieve a satisfactory CD4 cell count response.

## Supporting information

S1 Fig Flowchart showing the application of the study inclusion criteria. (TIF)

S2 Fig Cumulative incidence of a) serious AIDS or AIDS death (with SNAE or non-AIDS death as competing risk) and b) SNAE or non-AIDS death (with serious AIDS or AIDS death as competing risk) after baseline date by CD4 at ART initiation. Note: subfigures have different y-axis scale. Baseline is the date of ART initiation (“High” CD4 group) or the date of first reaching 500 CD4 cells/μl (“Low” CD4 group).
(EPS)

S3 Fig Cumulative incidence of a) fatal events (with non-fatal events as competing risk) and b) non-fatal events (with fatal events as competing risk) after baseline date by CD4 at ART initiation. Baseline is the date of ART initiation (“High” CD4 group) or the date of first reaching 500 CD4 cells/μl (“Low” CD4 group).
(EPS)

S4 Fig Cumulative incidence of a) serious AIDS or AIDS death (with all other types of events as competing risk), b) fatal or non-fatal CVD (with all other types of events as competing risk), c) fatal or non-fatal NADM (with all other types of events as competing risk) and d) Other fatal or non-fatal events (with all previous types of events as competing risk) after baseline date by CD4 at ART initiation. Baseline is the date of ART initiation (“High” CD4 group) or the date of first reaching 500 CD4 cells/μl (“Low” CD4 group).
(EPS)

S5 Fig Cumulative probability of death (without any competing risk and without censoring at the occurrence of any other event) by a) CD4 group (“High” i.e. those who started ART with 500+ and “Low” i.e. those who started ART with <500 CD4 cells/μl) and b) CD4 group with further stratification of the “Low” CD4 group according to CD4 at ART initiation. Baseline is the date of ART initiation (“High” CD4 group) or the date of first reaching 500 CD4 cells/μl (“Low” CD4 group).
(EPS)

S1 Appendix (DOCX)
