# Supplementary figures and images for: Low pre-ART CD4 count is associated with increased risk of clinical progression or death even after reaching 500 CD4 cells/μL on ART
Source: PLoS One. 2023 Mar 30;18(3):e0283648. doi: 10.1371/journal.pone.0283648 (PMC10062628; doi:10.1371/journal.pone.0283648)

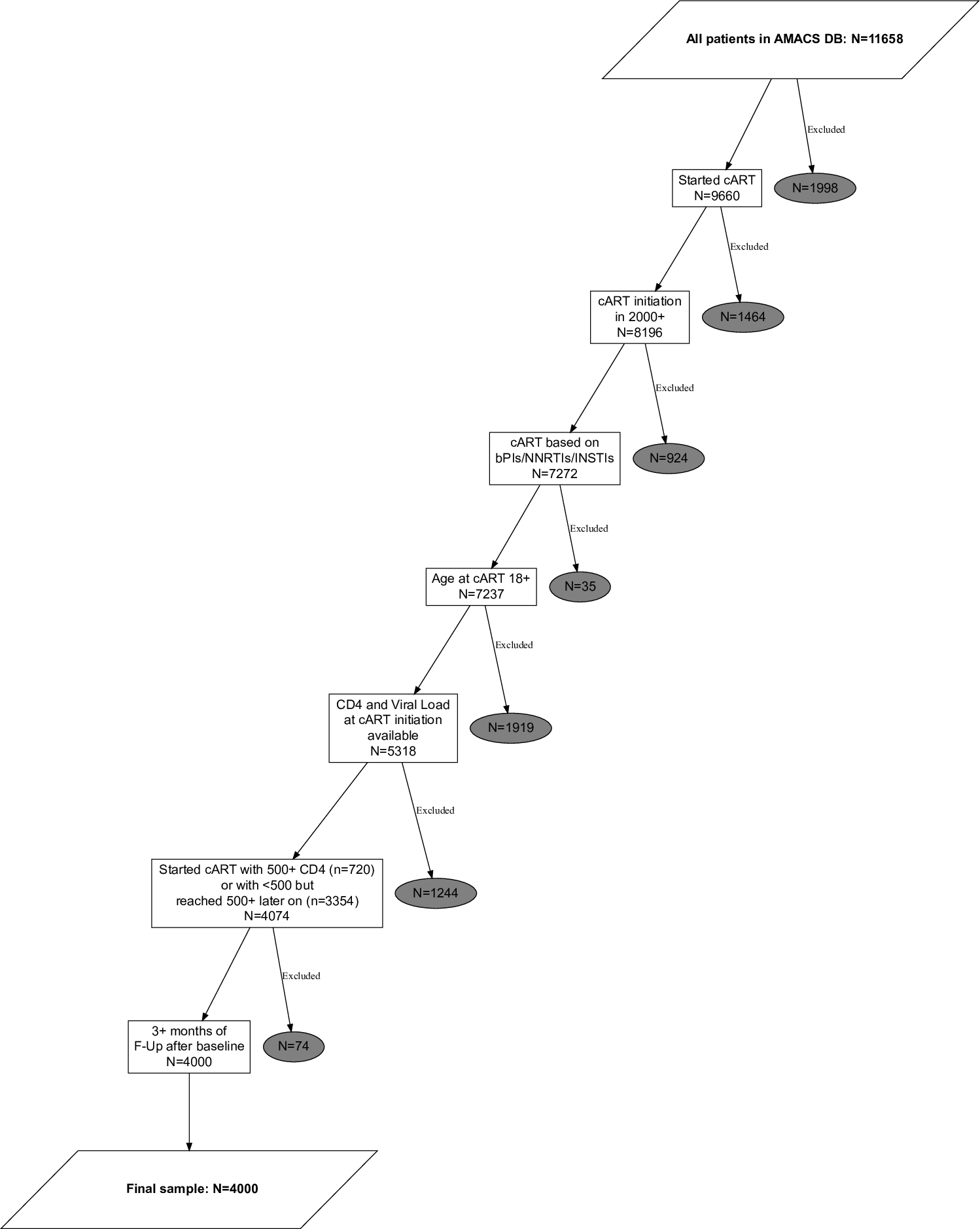

Supplement: S1 Fig — (TIF) [file pone.0283648.s001.tif]
