# Supplementary material for: Low pre-ART CD4 count is associated with increased risk of clinical progression or death even after reaching 500 CD4 cells/μL on ART
Source: PLoS One. 2023 Mar 30;18(3):e0283648. doi: 10.1371/journal.pone.0283648 (PMC10062628; doi:10.1371/journal.pone.0283648)
Supplement: S1 Appendix — (DOCX) [file pone.0283648.s006.docx]

**S1 Appendix**

**AMACS study**:

**Steering Committee**: Adamis G, Antoniadou A, Chini M, Chrysos G, Gikas A, Gogos HA, Katsarou O, Lazanas M, Metallidis S, Panagopoulos P, Paparizos V, Papastamopoulos V, Paraskevis D, Psychogiou M, Sambatakou H (Co-Chair), Sipsas NV, Touloumi G (Chair).

**Coordinating Center**:

Department of Hygiene, Epidemiology and Medical Statistics, Medical School, National and Kapodistrian University of Athens, Greece (Touloumi G, Pantazis N, Vourli G)

**Participating Centers**: 4th Dept of Internal Medicine, Medical School, National and Kapodistrian University of Athens, Attikon University Hospital (Antoniadou A, Papadopoulos A); Infectious Disease Unit, “Tzaneio” General Hospital of Piraeus (Chrysos G, Nitsotolis T); 1st Dept of Internal Medicine, Athens University, Medical School “Laikon” General Hospital (Psichogiou M); 1st Dept of Medicine, Infectious Diseases Unit, "G. Gennimatas" Athens General Hospital (Adamis G, Xylomenos G); 1st Dept of Internal Medicine, Infectious Diseases Section, Patras University Hospital (Gogos HA, Marangos MN); Blood Transfusion Unit, and National Reference Centre for Congenital Bleeding Disorders, Laikon General Hospital (Katsarou O, Kouramba A); Infectious Diseases Unit, Department of Pathophysiology, General Hospital of Athens “Laikon” and Medical School, National and Kapodistrian University of Athens, Athens, Greece (Sipsas NV, Kontos A); Infectious Diseases Unit, Red Cross General Hospital of Athens (Chini M, Lioni A); First Internal Medicine Department, Infectious Diseases Division, Medical School, Aristotle University of Thessaloniki  (Metallidis S, Tsachouridou O); AIDS Unit, Clinic of Venereologic & Dermatologic Diseases, Athens University, Medical School, Syngros Hospital (Paparizos V, Kourkounti S); HIV Unit, 2nd Dept. of Internal Medicine, Athens University, Medical School, Hippokration General Hospital (Sambatakou H); Infectious Diseases & HIV Division, Dept of Internal Medicine, Evaggelismos Athens General Hospital (Papastamopoulos V); Infectious Diseases Unit, University General Hospital of Alexandroupolis, Democritus University of Thrace (Panagopoulos P, Petrakis V); Department of Internal Medicine, University Hospital of Heraklion, Heraklion, Crete, Greece (Gikas A, Barbounakis E).

**Hellenic Society for the Study and Control of AIDS**: Lazanas M (Chair), Gogos H (Co-Chair)
